# Thymoquinone Loaded Topical Nanoemulgel for Wound Healing: Formulation Design and In-Vivo Evaluation

**DOI:** 10.3390/molecules26133863

**Published:** 2021-06-24

**Authors:** Mohammed S. Algahtani, Mohammad Zaki Ahmad, Ibrahim Ahmed Shaikh, Basel A. Abdel-Wahab, Ihab Hamed Nourein, Javed Ahmad

**Affiliations:** 1 Department of Pharmaceutics, College of Pharmacy, Najran University, Najran 11001, Saudi Arabia; msalqahtane@nu.edu.sa (M.S.A.); mzahmad@nu.edu.sa (M.Z.A.); 2 Department of Pharmacology, College of Pharmacy, Najran University, Najran 11001, Saudi Arabia; iashikh@nu.edu.sa (I.A.S.); babdelnaem@nu.edu.sa (B.A.A.-W.); 3 Department of Clinical Laboratory (Histopathology and Cytology), College of Applied Medical Sciences, Najran University, Najran 11001, Saudi Arabia; ihab213@gmail.com

**Keywords:** thymoquinone, black seed oil, ultrasonication, nanoemulgel, skin penetrability, silver sulfadiazine, wound healing

## Abstract

Thymoquinone is a natural bioactive with significant therapeutic activity against multiple ailments including wound healing. The poor aqueous solubility and low skin permeability limit its therapeutic efficacy. The present investigation aimed to improve the biopharmaceutical attributes of thymoquinone to enhance its topical efficacy in wound healing. A nanoemulsion-based hydrogel system was designed and characterized as a nanotechnology-mediated drug delivery approach to improve the therapeutic efficacy of thymoquinone, utilizing a high-energy emulsification technique. The black seed oil, as a natural home of thymoquinone, was utilized to improve the drug loading capacity of the developed nanoemulsion system and reduced the oil droplet size to <100 nm through ultrasonication. The influence of formulation composition, and the ultrasonication process conditions, were investigated on the mean globule size and polydispersity index of the generated nanoemulsion. Irrespective of surfactant/co-surfactant ratio and % concentration of surfactant/co-surfactant mixture, the ultrasonication time had a significant (*p* < 0.05) influence on the mean droplet size and polydispersity index of the generated nanoemulsion. The developed nanoemulgel system of thymoquinone demonstrated the pseudoplastic behavior with thixotropic properties, and this behavior is desirable for topical application. The nanoemulgel system of thymoquinone exhibited significant enhancement (*p* < 0.05) in skin penetrability and deposition characteristics after topical administration compared to the conventional hydrogel system. The developed nanoemulgel system of thymoquinone exhibited quicker and early healing in wounded Wistar rats compared to the conventional hydrogel of thymoquinone, while showing comparable healing efficacy with respect to marketed silver sulfadiazine (1%) cream. Furthermore, histopathology analysis of animals treated with a developed formulation system demonstrated the formation of the thick epidermal layer, papillary dermis along with the presence of extensive and organized collagen fibers in newly healed tissues. The outcome of this investigation signifies that topical delivery of thymoquinone through nanoemulgel system is a promising candidate which accelerates the process of wound healing in preclinical study.

## 1. Introduction

Physical injury to the skin that leads it to break and open is known as a wound [1]. Wound healing is one of the most complex physiological processes, which involve the events of clotting, coagulation, inflammation, and generation of new tissue [1,2]. Agents that help in the acceleration of wound healing are desirable to debride dead cell or tissue, minimize microbial infection, and enhance wound closure and healing [3]. Different therapeutics, through oral or parenteral routes, are utilized to treat the wound. However, systemic administration of a drug can result in many untoward effects [4].

Thymoquinone (TMQ) is a biologically active plant therapeutic, obtained from black seed (*Nigella sativa*), that has been shown to have multifunction properties, including antimicrobial [5,6], anti-inflammatory [7], anti-allergic [8], anti-oxidant [9], anti-neoplastic [10], anti-diabetic [11], and many more desirable properties [12,13] that have been demonstrated in different investigations. It has also been reported that TMQ significantly minimizes the histological tissue destruction caused by ischemia-reperfusion [14]. Additionally, TMQ has antimicrobial and resistance-modifying activities against pathogens [5,6]. Taking the consideration of properties mentioned above, Selcuk et al. (2013) studied the wound healing activity of TMQ in the rat model [15]. The wound healing properties of TMQ have been attributed due to its anti-microbial, resistance-modifying, anti-oxidant, and anti-inflammatory properties [5,6,7,8,9,10,16]. The therapeutic application of TMQ, as a wound-healing agent, has resulted in an enhanced anti-inflammatory response, diminished oxidative stress, better fibroblast formation, increased granular tissue production, augmented wound contraction, and re-epithelization [15,16]. The topical application of TMQ in wound healing is an effective approach as it offers direct access to the affected sites [17]. Despite its multifunction activity and varied promising medical application, the therapeutic effectiveness of TMQ is restricted because of poor water solubility and low skin penetrability that result in low systemic availability [11,18]. The major limiting factor that hindered the wound healing efficacy of TMQ is its poor aqueous solubility, low skin permeability, and photosensitivity [11,18]. An easy way for solving the associated stability and systemic availability issue of TMQ for better efficacy in wound healing is to assure its protection from photo-degradation, first-pass metabolism, and to enhance its targetability through topical administration. Topical delivery of TMQ can improve its local concentrations at the disease area, and control the drug release for improved efficacy of TMQ in wound healing.

Therefore, the design of an effective delivery system, which can ease the topical administration of TMQ through a nanoemulsion-based hydrogel system, will undoubtedly improve its therapeutic efficacy in wound healing. A nanoemulsion-based hydrogel system is an approach for nanotechnology-mediated drug delivery which is considered an efficient tool to enhance the biopharmaceutical attributes of poorly soluble drugs. The poorly water-soluble drug, such as TMQ, would be encapsulated into a lipophilic environment of an oil droplet of nano dimension, and stabilized through an optimized amount of surfactant/co-surfactant mixture along with an aqueous phase. To encapsulate the TMQ inside the oil droplet, black seed oil (natural home of TMQ) would be screened with a hypothesis of improvement in the drug loading and remain stable inside the natural environmental condition. This drug-loaded nanoemulsion system would be ultimately dispersed into a hydrogel system as a semisolid dosage form (called nanoemulgel) for topical application in wound healing. Hence, the present investigation aims to focus on the design and characterization of a nanoemulgel-based efficient formulation system of TMQ for topical application in wound healing.

## 2. Materials and Method

### 2.1. Materials

Thymoquinone (99%) was obtained from UFC Biotechnology, (Buffalo, NY, USA). Oleic acid, ethyl oleate, castor oil, isopropyl myristate, isopropyl alcohol, sesame oil, PEG 400, tween 20, tween 80, and Kolliphor EL, Solutol HS were purchased from Sigma Aldrich (Hamburg, Germany), Black seed oil was purchased from Amazing Herbs, (Buford GA, USA). Caproyl 90 and Transcutol HP were purchased from Gattefosse (Saint-Proest France), Carbopol-940 was obtained from Lubrizol (Wickliffe, OH, USA). All other excipients were of pharmaceutical-grade reagent.

### 2.2. Preformulation Investigation

#### 2.2.1. Solubility Study

Solubility of thymoquinone (TMQ) in different oil systems was determined by the shake-flask method [19,20,21,22,23]. Briefly, the weight amount of the drug was added to the specified oils (2 mL) in stopper vials and mixed with a vortex mixer. The sample was equilibrated in a water-shaker at 25 ± 0.5 °C for 48 h [24]. After that, samples were centrifuged (6000 rpm for 10 min), and the supernatant was filtered through a syringe filter of pore size 0.22 µm (Whatman^®^). The filtered sample was quantified to determine drug solubility using a UV-visible spectrophotometer (Perkin Elmer, Waltham, MA, USA) at 254 nm.

#### 2.2.2. Emulsification Efficiency

Emulsification efficiency of various surfactants and co-surfactant was determined by the method reported by Xi et al. (2009) with slight modification [19,20,21,24]. Briefly, oil in water (O/W) emulsion was prepared through shearing with the addition of a known amount of oil (5 µL) to the 5% aqueous dispersion system of surfactant and co-surfactant, through a vortex mixture, until turbidity appears [19,20,21]. The percentage transmittance (%T) of each emulsion was determined through UV-visible spectrophotometer analysis at 683.2 nm [19,20,21]. The aqueous dispersion system, having the ability to emulsify the maximum amount of the selected oil phase, was allowed to equilibrate and visually observe to remain as a homogenous system [19,20,21].

### 2.3. Preparation of Thymoquinone Loaded Nanoemulsion through Ultrasonication

The results obtained from the solubility study and emulsification efficiency investigation help to determine the oil phase and Smix phase (mixture of surfactant and co-surfactant) for the preparation of thymoquinone loaded nanoemulsion (TMQ-NE). A high-energy ultra-sonication technique was used to prepare TMQ-NE [25,26,27]. Initially, the coarse emulsion was prepared by mixing the 5% *w/w* (50 mg/g) of TMQ in the mixture of the oil phase and Smix through vortex mixture followed by addition of the aqueous phase with continuous vortexing for 1 min [22]. The generated coarse emulsion phase was ultrasonicated (Ultrasonic Homogenizer, FS-300N, Zhejiang, China) further in a water bath for a different time interval (3, 5, and 10 min) at an ultrasonication amplitude of 40% [26,27]. Eighteen formulations of different compositions were developed and evaluated to select the optimum formulation of TMQ-NE.

### 2.4. Characterization of Thymoquinone Loaded Nanoemulsion

Initially, TMQ-NE formulations were prepared in triplicate and evaluated for thermodynamic stability study, droplet size distribution and polydispersity index (PdI), zeta potential, viscosity, and drug content.

#### 2.4.1. Thermodynamic Stability Study

The developed TMQ-NE were subjected to thermodynamic stability assessments to determine and exclude metastable/unstable formulation composition from a further investigation [28]. These investigations were carried out through heating-cooling cycles, centrifugation and freeze-thaw cycles, according to the reported method, by our group, in previous investigations [19,20,21].

#### 2.4.2. Analysis of Droplet Size, Polydispersity Index, and Zeta Potential

The mean globule size, and PdI, of developed TMQ-NE were analyzed at 25 °C by photon correlation spectroscopy using a Zetasizer Nano ZS90 (Malvern Instruments, Malvern, UK) [29,30]. The zeta potential (*ζ*) of TMQ-NE was also determined using the same instrument.

#### 2.4.3. Determination of Viscosity

The viscosity of the optimized TMQ-NE was analyzed, without dilution, using a Bohlin rotational viscometer [19,20,21,31].

#### 2.4.4. Analysis of Drug Content

TMQ content in the optimized TMQ-NE formulations was determined by diluting 100 μL of TMQ-NE 1000 times [19,20,21,32] with methanol and quantifying the content of TMQ, using an UV-visible spectrophotometer, at λmax at 254 nm.

### 2.5. In-Vitro Drug Release

The formulations, which pass the thermodynamic stability, were chosen for the in-vitro drug release investigation through the dialysis bag technique [19,20,21,33]. Dialysis bags (12–14 kDa) were filled with 1 mL of each TMQ-NE formulation and suspended in phosphate buffer solution (PBS) as a release medium (pH 7.4) at 37 ± 0.5 °C. At specified time intervals, 1 mL aliquots were taken out and replaced by the same volume of PBS. The amount of TMQ in the aliquots was quantified by UV-spectroscopy at λmax 254 nm. These experiments were carried out in triplicate.

### 2.6. Preparation and Characterization of TMQ Nano-Emulgel

The known amounts of Carbopol 940 were uniformly dispersed into a specified volume of water. The selected TMQ-NE formulation was incorporated with Carbopol 940 to give a final concentration of 0.5% (*w/w*) of TMQ nanoemugel (TMQ-NEG) for topical administration [19,20,21,22]. Five percent glycerin was added as a humectant into the dispersion system [19,20,21]. It will provide soothing effects. Triethanolamine was added to the formulation in a drop-by-drop, which resulted in instant conversion to a hydrogel system at pH 5.5 [19,20,21]. The pH, rheology, spreadability, and drug content uniformity of the TMQ-NEG were assessed by the method previously reported by our group [19,20,21,22]. Local accumulation efficiency (LAE) of the TMQ-NEG and TMQ-gel was obtained as the ratio of TMQ accumulated on the skin to that of TMQ permeated through the skin [34,35,36].

### 2.7. Ex-Vivo Skin Permeability

Skin permeability study of the TMQ-NEG was performed as per the method described by our groups in the previously reported studies [19,20,21], utilizing Franz-Diffusion cell. Shaved, excised dorsal skin sample from Wistar rat was placed between the donor and receptor compartments. The TMQ-NEG was (500 mg) was kept in the donor compartment, and the receptor compartment was filled with phosphate buffer of pH 7.4 [21]. The whole assembly was maintained at 37 °C under a magnetic stirrer. The aliquot sample of 1 mL was withdrawn at a different time interval (0, 0.5, 1, 2, 3, 4, 6, 8, 10, and 12 h) and replaced with the same volume of fresh media [19]. The aliquot was suitably diluted and quantified using UV-spectrophotometer at λmax 254 nm.

The drug deposition in rat skin was estimated on the same skin by the tape stripping technique [20]. After the 12 h of ex-vivo skin permeability study, the skin sample was unclipped from the assembly and washed with a phosphate buffer. Tape stripping was performed using cellophane tape. First strips were discarded to avoid the drug adhering to the skin surface. The next 15 strips were used for the removal of the subcutaneous layer [19,20]. The treated skin sample, and stripped tape, were chopped and incubated in ethanol for the complete extraction of the drug [21]. The incubated sample was sonicated for 5 min and then centrifuged. The extracted sample was analyzed using an UV-spectrophotometer at λmax 254 nm to measure the amount of drug deposited in the skin. The same is repeated to estimate the skin permeation and deposition characteristics of TMQ using TMQ-gel (accurately weighed quantity of TMQ was solubilized in the small volume of propylene glycol and uniformly dispersed into placebo gel to obtain TMQ-gel formulation of strength 0.5% *w/w*). The obtained results are compared to know the skin permeation, and deposition characteristics, of TMQ with respect to developed TMQ-NEG formulation.

### 2.8. In-Vivo Animal Study

#### 2.8.1. Experimental Protocol

The experimental protocol to carry out the wound healing activity in a Wistar rat was approved by the institutional ethical committee (Najran University, KSA) and followed their guidelines to perform the study (Ref. No: 25-01-01-20-EC). Wistar rats of weight 200–250 g were used for the wound healing investigation. The Wistar rats were housed in polypropylene cages, with free access to a standard laboratory diet and water ad libitum. The animals were kept under standard laboratory conditions (25 ± 2 °C and 55 ± 5% RH). The rats were anesthetized under aseptic conditions (50 mg/kg intraperitoneal injection of Ketamine HCl) [37]. Part of their backside was shaved and a deep wound area (2.0 cm^2^) was created using a sterile biopsy punch (Acu punch, Acuderma Inc., Louderale, FL, USA). All animals were divided into four groups with 4 rats in each group. Group I consists of the negative control group with no treatment, group II was treated with marketed preparation of 1% *w/w* silver sulfadiazine cream (quantity applied-100 mg) twice a day, group III was treated with 0.5% TMQ-gel (quantity applied = 250 mg) twice a day and group IV was treated with 0.5% TMQ-NEG (quantity applied = 250 mg) twice a day. All the treatment groups (Group II, III, and IV) received the therapy for 20 consecutive days.

#### 2.8.2. Assessment of Wound Healing Area

Assessment of wound healing area was carried out in terms of wound contraction percentage, epithelization period, and wound closure time [38,39]. The percentage of wound contraction was calculated using the following formula (initial size of the wound considered as 100%) [38,39].

(1) $$\%wound\;contraction=\frac{\left(Initial\;wound\;area-Specific\;day\;wound\;area\right)}{Initial\;day\;wound\;area}\times 100$$

#### 2.8.3. Histopathology

On the last day of the wound healing experiment, the animals were anesthetized using Ketamine HCl (50 mg/kg, i.p.), euthanized, and specimens of wound tissue with the adjacent healthy tissue were collected. The collected samples were fixed in 10% formalin and were subjected to routine histopathological tissue examination. The wound tissue specimen was sectioned with a microtome (Leica RM 2245) and then stained with hematoxylin-eosin. The prepared tissue slide was examined under a light microscope. To evaluate the collagen content, the wound tissue specimen was sectioned using a microtome, stained with Van Gieson stain for collagen fiber, and examined under a microscope (Leica DM IRM, Leica Microsystems, Wetzlar, Germany).

### 2.9. Statistical Analysis

The statistical analysis was carried out using SPSS software (version 23 SPSS Inc, Chicago, IL, USA). The obtained data were analyzed utilizing one-way ANOVA followed by Tukey’s multiple comparisons tests. The *p* < 0.05 was considered statistically significant.

## 3. Results and Discussion

### 3.1. Preformulation Investigation

TMQ loaded NE was developed by exploiting various pharmaceutically acceptable NE components using the ultra-sonication technique. The Smix system, used in this formulation, has enhanced the extent of nanoemulsification; besides, it was expected to enhance the permeation of TMQ across the skin [31]. The solubility of TMQ in different oils was evaluated, including oleic acid, ethyl oleate, black seed oil, castor oil, sesame oil, castor oil, capryol 90, and isopropyl alcohol. TMQ demonstrated the higher solubility in black seed oil (>500 mg/mL), followed by capryol 90 (>450 mg/mL), ethyl oleate (300.45 ± 1.82 mg/mL), oleic acid (249.49 ± 1.11 mg/mL), and castor oil (99.38 ± 1.79 mg/mL). After considering the solubility and therapeutic properties, black seed oil was selected to develop TMQ-NE.

**Figure 1:**
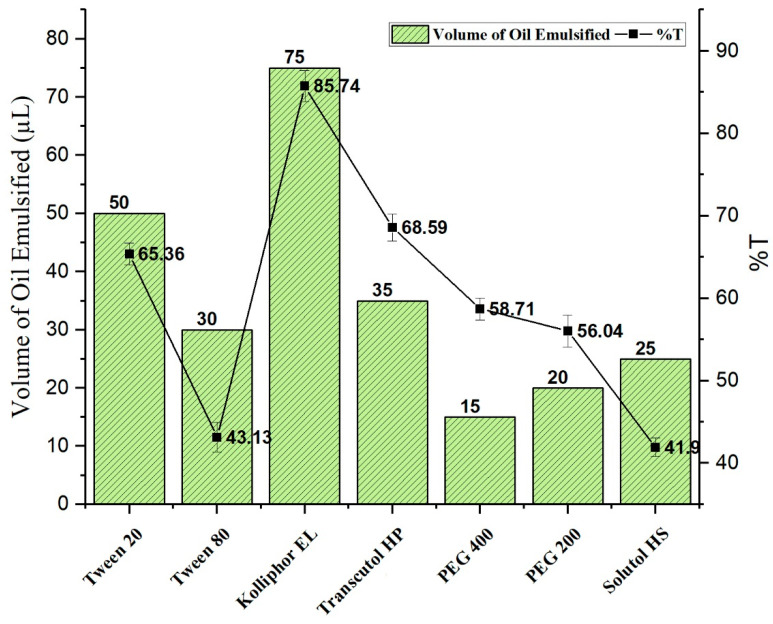
Emulsification efficiency of different surfactant and co-surfactant.

Kolliphor EL, Tween 80, Tween 20, Solutol HS, Transcutol HP, PEG 400, and PEG 200 were investigated as surfactant and co-surfactant phase to design and develop TMQ-NE (Figure 1). Kolliphor EL being a surfactant demonstrated the maximum emulsification efficiency (%T with 85.74 ± 1.35), while Transcutol HP being a co-surfactant exhibited the maximum emulsification efficiency (%T with 68.59 ± 1.349). Based on the results, a mixture of Kolliphor EL and Transcutol HP was selected as the Smix phase.

### 3.2. Preparation of Nanoemulsion

**Figure 2:**
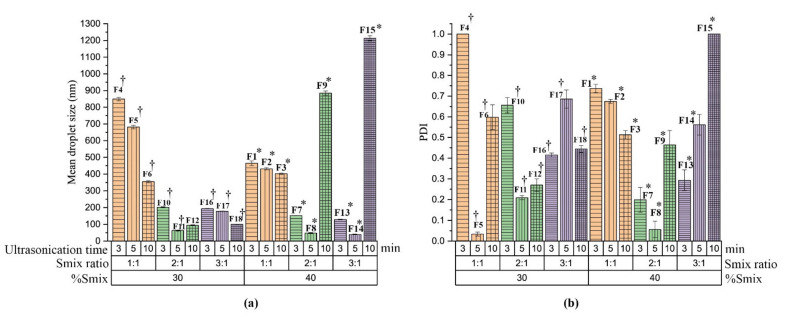
The influence of formulation composition of F1-F14 (Smix ratio, and %concentration of Smix) and the ultrasonication process conditions (ultrasonication time) on the mean droplet size and PdI. (**a**) Mean droplet size of NE prepared by ultrasonication at an amplitude of 40% at a different time interval (3, 5, and 10 min) with different Smix ratios (1:1, 2:1, and 3:1) and Smix concentration at 30, and 40%. (**b**) PdI of NE prepared by ultrasonication at an amplitude of 40% at a different time interval (3, 5, and 10 min) with different Smix ratios (1:1, 2:1, and 3:1) and Smix concentration at 30, and 40%. ^†^ represents formulation compositions as %oil, %Smix, and %water are 10%, 30%, and 60% respectively while * represents formulation compositions as % oil, % Smix, and %water are 10%, 40%, and 50%, respectively.

Initially, eighteen TMQ-NE formulations were prepared by the ultrasonication method, by varying the ingredient composition and process parameters, as shown in Figure 2. All NE were prepared in triplicate. From the eighteen formulations, five stable formulations were selected for further characterization studies (F8, F11, F12, F14, and F18) for having the least mean droplet size (≤100 nm) at the applied process conditions (3, 5, and 10 min).

The Smix concentration, and the Smix ratio, had a noticeable influence on the mean droplet size and PdI (*p* < 0.05) of NE produced (Figure 2). In general, the increase in the Smix concentration decreases the mean droplet size of NE. The Smix at the ratio of 1:1 concentration produced NE with a larger mean droplet size (355 ± 6.56 nm to 849.3 ± 10.17 nm) Figure 2). For Smix of a 2:1 ratio, the NEs produced have a lower mean droplet size (202.1 ± 2.72 nm to 48.45 ± 0.74 nm) except at the concentration of 40% Smix (2:1) with 10 min ultrasonication time (mean droplet size = 884.9 ± 12.66 nm) (Figure 2). The same pattern was observed for Smix 3:1 (193.6 ± 1.09 nm to 40.02 ± 0.83 nm) except for the 40% Smix (3:1) with 10 min ultrasonication time (Figure 2).

Irrespective of the Smix ratio and % concentration of Smix, the ultrasonication time had a significant (*p* < 0.05) influence on the mean droplet size and PdI of the generated NE. As the ultrasonication time increases from 3 to 5 min, the mean droplet size decreases. However, at 10 min, the mean droplet size increases, particularly in the case of Smix ratio 2:1. The PdI was found significantly (*p* < 0.05) higher at all process conditions at the Smix ratio of 1:1 and with a 30% concentration of Smix. The excess exposure to the ultrasonication energy, known as over-processing, results in intense turbulence, which promotes the collision between NE globules, their coalescence forming a larger globular size [40,41].

From the total of eighteen NE formulations, F8, F11, F12, F14, and F18 (Figure 2) show mean droplet size of less than 100 nm. The average globules size of five selected formulation varies between 40.02 ± 0.83 and 99.66 ± 1.43 nm, and the polydispersity index varies between 0.052 ± 0.004 to 0.542 ± 0.05. Those NE formulations were selected for further characterization studies.

### 3.3. Characterization of Nanoemulsion

#### 3.3.1. Thermodynamic Stability Study

The results of thermodynamic stability are presented in Table 1. The thermodynamic stability of any system is governed by the change in free energy between the system and its milieu [42,43]. The thermodynamic stability tests were conducted to identify the presence of any metastable NE in the screened formulation. All five formulations were found to be stable when subjected to the heating-cooling cycle, centrifugation, and freeze-thaw cycle. This stability could be correlated to the zeta potential of the developed formulations, which varies between −26.7 and −30.6 mV. The stability of any NE has a direct relation to the magnitude of the surface charge. It has been demonstrated that high repulsive force between the droplets of NE prevents the coalescence. Otherwise, the system may be unstable and could result in phase separation [44].

#### 3.3.2. Viscosity

The viscosity measurement of five prepared TMQ-NE (F8, F11, F12, F14, and F18) was carried out at an ambient temperature (25 °C). F14 shows the maximum viscosity (88.82 ± 1.27) whereas F12 exhibits minimum viscosity (71.04 ± 1.02 mPas). This variation is proportionate to the % concentration of surfactant in NE formulation.

#### 3.3.3. Analysis of Drug Content

The drug content analysis in the selected five TMQ-NE (F8, F11, F12, F14, and F18) formulations were performed by the UV-visible spectrophotometer. The % content of TMQ, in different TMQ-NE formulations, varies from 98.74 ± 0.445 to 99.32 ± 0.119.

The ideal droplet size of NEs, for topical administration, should be near 50 nm, with a PdI value less than 1. This will provide a larger surface area for skin permeability and lead to deeper skin penetration of encapsulated drugs [19,20]. Considering this, the F8, F11, and F14 formulation systems of TMQ-NE (having droplet size near to 50 nm with PdI value <1, as shown in Supplementary Figures S1–S3) were chosen to investigate the in-vitro drug release profile.

### 3.4. In-Vitro Drug Release

**Figure 3:**
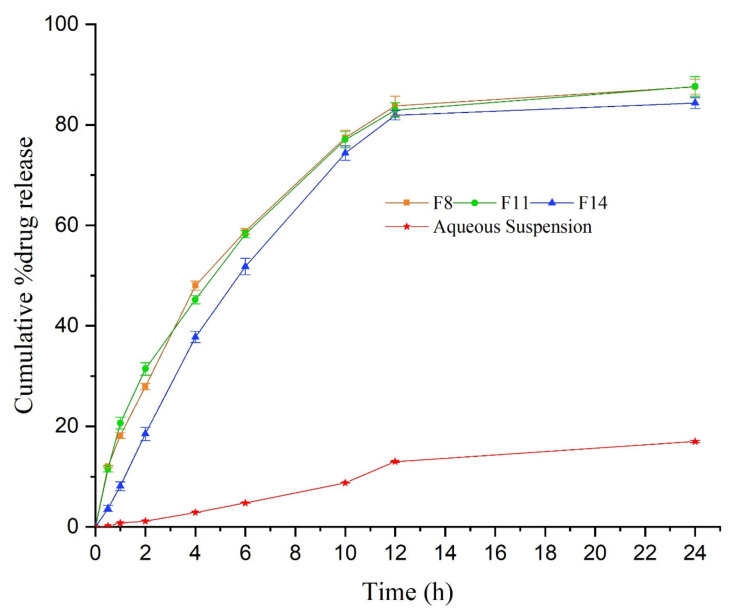
In-vitro drug release from TMQ-loaded NE system.

The in-vitro release of TMQ from the screened formulation (F8, F11, and F14) is shown in Figure 3. The in-vitro release study was carried out for 24 h. After 12 h, nearly 80% of the drug was released from all the screened formulations. The maximum amount of the drug released from F8, F11, and F14 were 87.1 ± 1.49%, 86.65 ± 1.97%, and 84.3 ± 1.06%, respectively, after 24 h. The in-vitro release of TMQ from the screened TMQ-NE formulation was compared to the in-vitro release of pure TMQ as an aqueous suspension. The in-vitro release of TMQ from all screened TMQ-NE formulations has significantly (*p* < 0.05) exceeded the in-vitro release of TMQ from the aqueous suspension. The formulation with maximum drug release, and minimum concentration of Smix (F11), was optimized to convert into a NEG system for topical application.

### 3.5. Preparation and Characterization of Nano-Emulgel

Although the optimized NE formulation (F11) was in the nanosize range, their low viscosity, due to their liquid state, impedes their topical application for dermal use. Therefore, the selected NE (F12) was uniformly dispersed into the gel matrix of carbopol 940 to give the final concentration of 0.5% TMQ in TMQ-NEG with the desired consistency to be applied topically for a more patient-friendly application. The pH, rheology, spreadability, and drug content uniformity of the developed TMQ-NEG were determined and analyzed.

**Figure 4:**
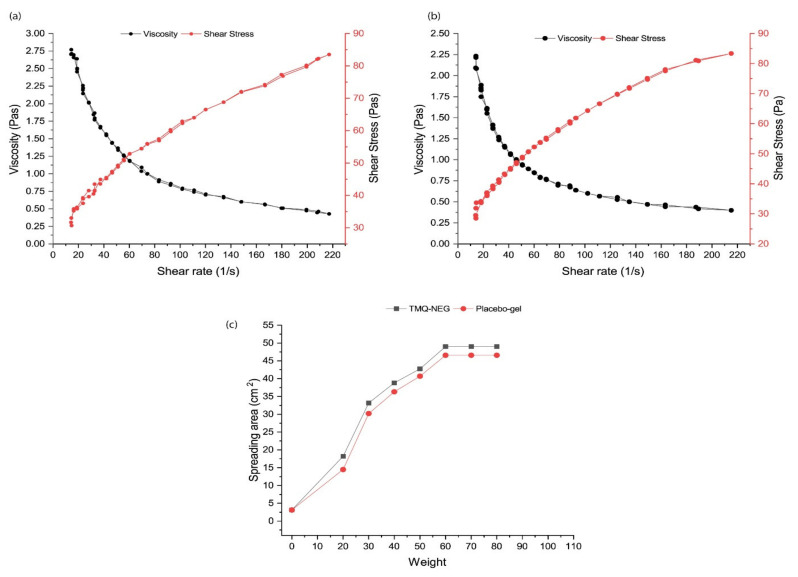
Rheology profile of gel (**a**) TMQ-NEG (**b**) Placebo gel (**c**) Spreadability behavior of TMQ-NEG and placebo gel.

The pH range of the developed TMQ-NEG was found to be the range of acid mantle pH of the skin (5.53 ± 0.04), permitting the topical use of the developed formulation [19,20,21]. The rheological characteristics of the topically applied formulations govern their spreading, extrudability, and drug release [45,46]. The rheological behavior of developed TMQ-NEG and placebo carbopol-940 gel are graphically depicted in Figure 4a,b. The prepared TMQ-NEG demonstrated a similar rheological behavior as compared to placebo gel, and the incorporated TMQ-NE did not affect its rheology. The developed TMQ-NEG demonstrated the pseudoplastic behavior with thixotropic properties, and this behavior is desirable for topical application [45,46]. The developed TMQ-NEG demonstrated excellent spreadability behavior suitable for the topical application to wounded skin. The spreading area of the gel increases, proportionally, with the applied force in the form of weight (Figure 4c). The TMQ was uniformly distributed throughout the NEG system with a % uniformity of 99.01 ± 0.211, and the drug loss was minimum during formulation development. 

### 3.6. Ex-Vivo Skin Permeability

A comparative ex-vivo drug skin permeability, and drug deposition, study was performed employing Franz-diffusion cell on the excised skin of Wistar rat for TMQ-NEG and TMQ-gel system. The results obtained from ex-vivo drug skin permeability and drug deposition are presented in Table 2. The skin deposition of TMQ, from TMQ-NEG and TMQ-gel, were found to be 965.65 ± 12.84 µg/cm^2^ and 150.93 ± 1.80 µg/cm^2^, respectively. The percutaneous drug flux (J) of the TMQ, from the TMQ-NEG, was approx. five folds (23.14 ± 0.22) of the TMQ from the TMQ-gel (4.78 ± 0.08). The permeability coefficient (K_p_ × 10^−3^) of the TMQ, from the TMQ-NEG, was also approx. five folds (4.88) of the TMQ from the TMQ-gel (1.91). The permeation enhancement ratio (ER) of TMQ, released from the TMQ-NEG, was 4.83. LAE of TMQ-NEG and TMQ-gel was 1.76 ± 0.015 and 1.25 ± 0.03, respectively.

This investigation was carried out to demonstrate the skin permeation potential of TMQ-NEG in comparison to TMQ-gel formulation. It has been reported that NEG increases the permeation of loaded drugs in the deep skin layer and decreases the lag time [45]. The cumulative amount of TMQ permeated in the ex-vivo skin permeability study was used to evaluate the various permeation parameters and to establish the comparative skin permeability profile of TMQ. TMQ-NEG demonstrated the enhanced cumulative permeation of the drug (549.16 ± 3.10 µg/cm^2^) as compared to TMQ-gel (120.75 ± 2.43). This may be due to the presence of Smix in the composition of the developed formulation system. Here, transcutol HP was used as a co-surfactant which helps in enhanced permeation of the drug across the skin [47]. Furthermore, skin permeation of TMQ-NEG formulation (965.65 ± 12.84 µg/cm^2^) was significantly higher than TMQ-gel formulation (150.93 ± 1.80 µg/cm^2^). The LAE value of TMQ-NEG was higher by a factor of 1.4 than that of TMQ-gel, suggesting a higher accumulation of the drug in the skin for local action [36]. It is interesting to note that TMQ-NEG has higher skin permeation as compared to TMQ-gel. However, the best LAE values are always obtained with the formulation of a negatively charged surface [36]. Here, in our case, negative zeta potential plays a crucial role in LAE.

### 3.7. In-Vivo Wound Healing Study

**Figure 5:**
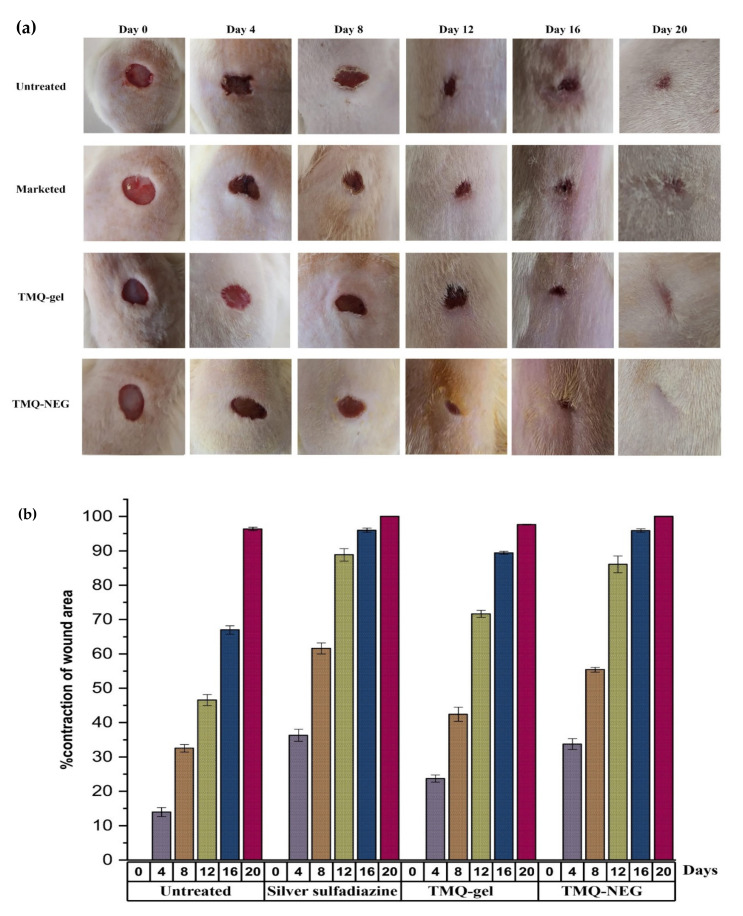
(**a**) In-vivo wound healing study in Wistar rat (**b**) percentage contraction of wound area as an evaluation parameter for in-vivo wound healing activity of marketed silver sulfadiazine cream, TMQ-gel, and TMQ-NEG in the Wistar rat.

TMQ has therapeutic potential for wound healing activity [15,16,48]. Here, TMQ-NEG was formulated to enhance the wound healing effects of TMQ through the NEG system. Figure 5a shows the in-vivo wound healing effect of the topically applied TMQ-NEG on the Wistar rat model (group IV) in comparison to animals treated with standard 1% *w/w* silver sulfadiazine cream (group II) and TMQ-gel (group III). The contraction of the wound area was monitored at different time intervals i.e., 0, 4th, 8th, 12th, 16th^,^ and 20th day (Figure 5b). On day four of post-wounding, a hard thrombus swelling and exudates were observed on the wound area of group I rats. While, in other groups, comparatively soft thrombus with a decrease in inflammation, and no discharge, was observed with group IV, followed by group II and group III. On day eight, reddish connective tissue was formed. The formation of this granulation tissue was observed in animals of group I and group III. However, it was observed earlier (on the 6th day of post wounding) in animals of group II and group IV. The animals treated with 1% silver sulfadiazine cream, TMQ-gel, and TMQ-NEG significantly (*p* < 0.05) enhanced the wound healing effects on rat models and helped in the contraction of wound area from day 4 to 20 in comparison to the untreated animal group. The complete epithelization time for the animal under control (untreated) was 16.6 ± 0.57 days, while the animals of treated groups with the marketed silver sulfadiazine cream, TMQ-gel, and TMQ-NEG were 11.66 ± 1.52, 14.33 ± 0.57, and 10.33 ± 0.57 days, respectively. The complete epithelization period was significantly (*p* < 0.05) smaller in TMQ-NEG and 1% silver sulfadiazine group as compared to the untreated group. It was observed that TMQ-NEG showed a comparable wound healing effect with respect to the 1% silver sulfadiazine cream (Figure 5a,b). 

**Figure 6:**
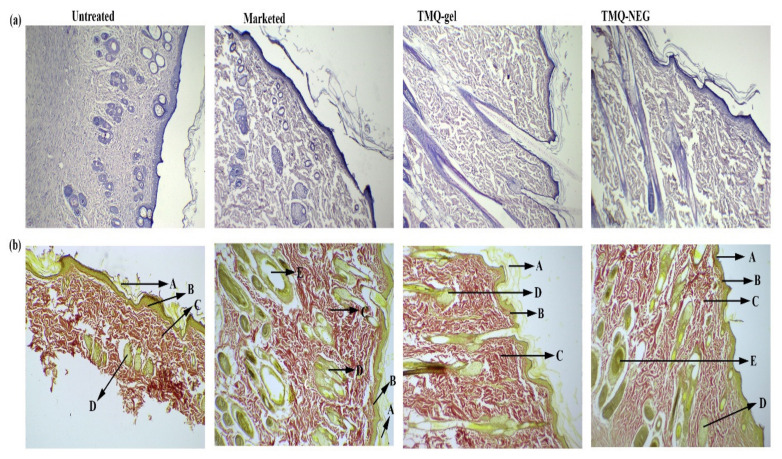
Histopathology analysis of newly healed tissue at day 20 (**a**) stained with hematoxylin-eosin (**b**) stained with Van Gieson to observe collagen formation (at 10× magnification). A—stratum corneum; B—papillary dermis; C—collagen fibers; D—sebaceous gland; E—hair follicles.

Histopathological analysis for the formation of collagen, re-epithelialization, and inflammation was performed on day 20. The histopathological finding of wound healing skin, for the different treatment groups, is presented in Figure 6. Comparatively large amounts of granulation tissue and fewer mononuclear inflammatory cells were observed in TMQ-NEG treated group, followed by animals treated with 1% silver sulfadiazine and TMQ-gel.

As shown in Figure 6, the presence of extensive, and organized, collagen fiber [49] was observed in animals treated with TMQ-NEG and 1% silver sulfadiazine. Furthermore, the TMQ-NEG treated group demonstrated the formation of the thick epidermal layer [49], papillary dermis [49], sebaceous glands [50], and hair follicles [50], followed by the animal treated with marketed cream of 1% silver sulfadiazine. No sign of inflammation was observed on the healed tissue of animals treated with TMQ-NEG and marketed cream of 1% silver sulfadiazine. The deeper penetrability of TMQ, through the nanoemulgel formulation system, augments the formation of extensive, and organized, cellular structure in newly healed skin tissues compared to the untreated group and animals treated with TMQ-gel.

## 4. Conclusions

A nanoemulsion-based hydrogel system was successfully developed and assessed to observe the improvement in the therapeutic efficacy of TMQ in wound healing. The high solubilization of TMQ in black seed oil was helpful to encapsulate the solubilized drug in nanosized oil droplets with high loading, exploiting ultrasonication technique. The oil-in-water nature of developed nanoemulsions was helpful to uniformly distribute into a cross-linked matrix of an aqueous hydrogel system and significantly enhanced the skin penetrability of TMQ in deeper tissue on topical application. This nanotechnology-mediated drug delivery approach helps to improve the therapeutic efficacy of TMQ in wound healing investigations in Wistar rats. The histopathology analysis of newly healed tissues revealed that deeper penetrability of TMQ through a nanoemulgel formulation system was helpful to form extensive, and organized, cellular structure in newly healed skin compared to conventional hydrogel formulation of TMQ.

## Figures and Tables

**Table 1:** Characterization of selected TMQ-loaded NE for thermodynamic stability, droplet size distribution, zeta potential, % drug content, and viscosity.

Formulation Code	Thermodynamic Stability			Mean Droplet Size (nm)	PdI	Zeta Potential (mV)	Drug Content (%)	Viscosity (mPas)
	Heating Cooling Cycle	Centrifugation Study	Freeze-Thaw Cycle					
F8	√	√	√	48.45 ± 0.74	0.052 ± 0.004	−29.5 ± 0.30	99.32 ± 0.119	77.81 ± 1.55
F11	√	√	√	64.22 ± 0.94	0.203 ± 0.01	−30.6 ± 0.40	99.14 ± 0.112	74.91 ± 1.74
F12	√	√	√	94.67 ± 0.71	0.26 ± 0.03	−30.5 ± 0.30	98.74 ± 0.445	71.04 ± 1.02
F14	√	√	√	40.02 ± 0.83	0.542 ± 0.05	−26.7 ± 0.26	99.09 ± 0.49	88.82 ± 1.27
F18	√	√	√	99.66 ± 1.43	0.428 ± 0.017	−28.9 ± 0.25	99.04 ± 0.258	85.38 ± 2.25

**Table 2:** Characterization of TMQ-loaded NEG to determine skin penetrability profile compared to TMQ-gel.

Formulation	The Cumulative Amount of Drug Permeated (µg/cm^2^)	Drug Deposited in the Skin (µg/cm^2^)	Lag Time (h)	Flux (µg/cm^2^·h)	Permeability Coefficient (K × 10^−3^)	Local Accumulation Efficiency (LAE)
TMQ-NEG	549.16 ± 3.10	965.65 ± 12.84	0.89 ± 0.01	23.14 ± 0.22	9.26 ± 0.09	1.76 ± 0.015
TMQ-gel	120.75 ± 2.43	150.93 ± 1.80	2.09 ± 0.04	4.78 ± 0.08	1.91 ± 0.03	1.25 ± 0.03

## Data Availability

The data presented in this study are available in article or Supplementary Materials.

## Supplementary Materials

The following are available online, Figure S1: Droplet size (14.07 nm with PdI 0.056) and zeta potential (−29.2 mV) of TMQ-NE [F8]. Figure S2: Droplet size (63.28 nm with PdI 0.209) and zeta potential (−30.2 mV) of TMQ-NE [F11]. Figure S3: Droplet size (39.12 nm with PdI 0.562) and zeta potential (−26.8 mV) of TMQ-NE [F14].
