# Supplementary material for: Thymoquinone Loaded Topical Nanoemulgel for Wound Healing: Formulation Design and In-Vivo Evaluation
Source: Molecules. 2021 Jun 24;26(13):3863. doi: 10.3390/molecules26133863 (PMC8270244; doi:10.3390/molecules26133863)
Supplement: Supplementary file 1 [file molecules-26-03863-s001.zip › molecules-1261171-supplementary.pdf]

| | | | Diam. (nm) | % Intensity | Width (nm) |
|---|---|---|---|---|---|
| Z-Average (r.nm): | 48.07 | Peak 1: | 51.44 | 100.0 | 13.90 |
| PdI: | 0.056 | Peak 2: | 0.000 | 0.0 | 0.000 |
| Intercept: | 0.934 | Peak 3: | 0.000 | 0.0 | 0.000 |
| Result quality : | Good | | | | |

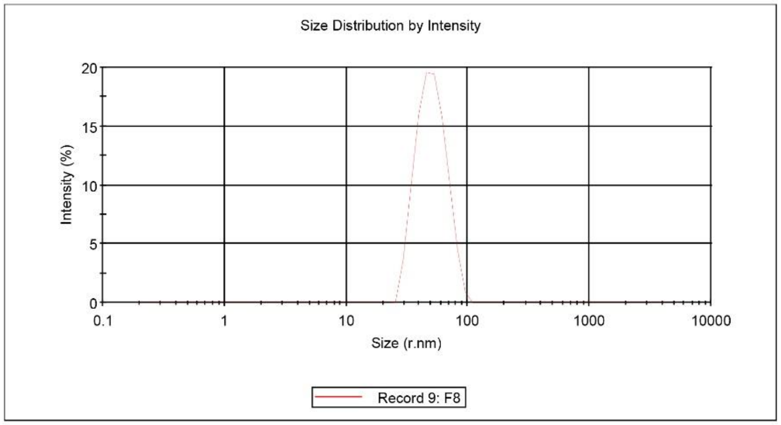


| | | | Mean (mV) | Area (%) | St Dev (mV) |
|---|---|---|---|---|---|
| Zeta Potential (mV): | -29.2 | Peak 1: | -29.2 | 100.0 | 5.41 |
| Zeta Deviation (mV): | 5.41 | Peak 2: | 0.00 | 0.0 | 0.00 |
| Conductivity (mS/cm): | 0.0133 | Peak 3: | 0.00 | 0.0 | 0.00 |
| Result quality : | Good | | | | |

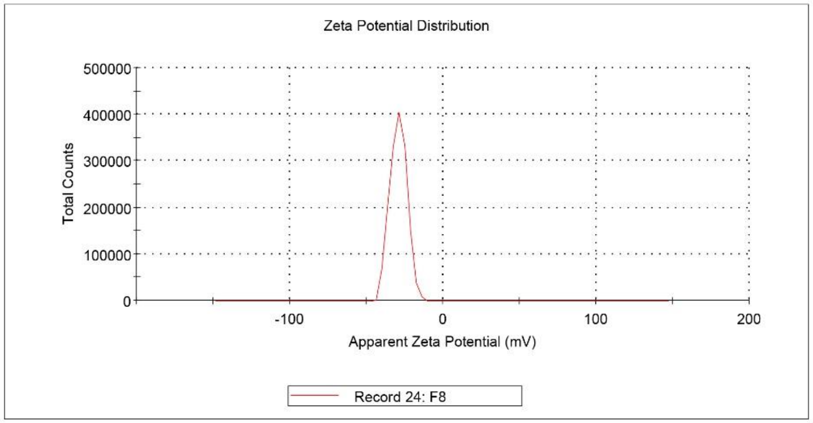


**Figure S1:** Droplet size (*14.07 nm with PdI 0.056*) and zeta potential (-*29.2 mV*) of TMQ-NE [F8]

| | | | Diam. (nm) | % Intensity | Width (nm) |
|---|---|---|---|---|---|
| Z-Average (r.nm): | 63.28 | Peak 1: | 69.01 | 100.0 | 18.74 |
| PdI: | 0.209 | Peak 2: | 0.000 | 0.0 | 0.000 |
| Intercept: | 0.923 | Peak 3: | 0.000 | 0.0 | 0.000 |
| Result quality : | Good | | | | |

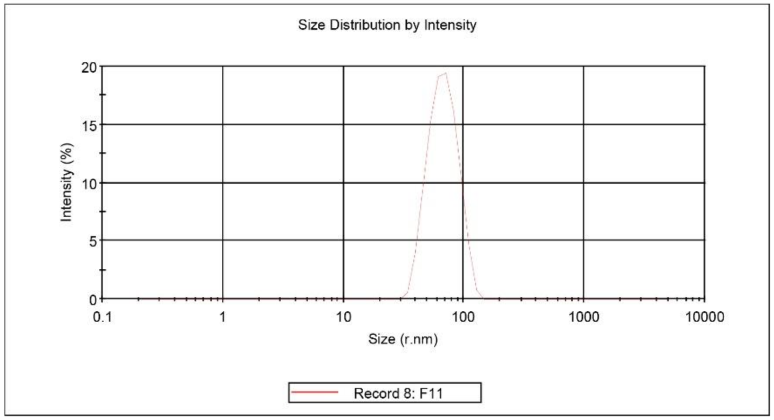


| | | | Mean (mV) | Area (%) | Width (mV) |
|---|---|---|---|---|---|
| Zeta Potential (mV): | -30.2 | Peak 1: | -30.2 | 100.0 | 5.15 |
| Zeta Deviation (mV): | 5.15 | Peak 2: | 0.00 | 0.0 | 0.00 |
| Conductivity (mS/cm): | 0.00807 | Peak 3: | 0.00 | 0.0 | 0.00 |
| Result quality : | Good | | | | |

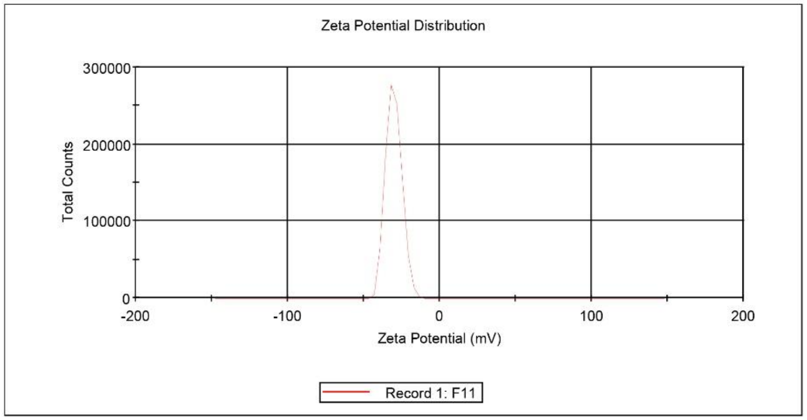


**Figure S2:** Droplet size (*63.28 nm with PdI 0.209*) and zeta potential (-*30.2 mV*) of TMQ-NE [F11]

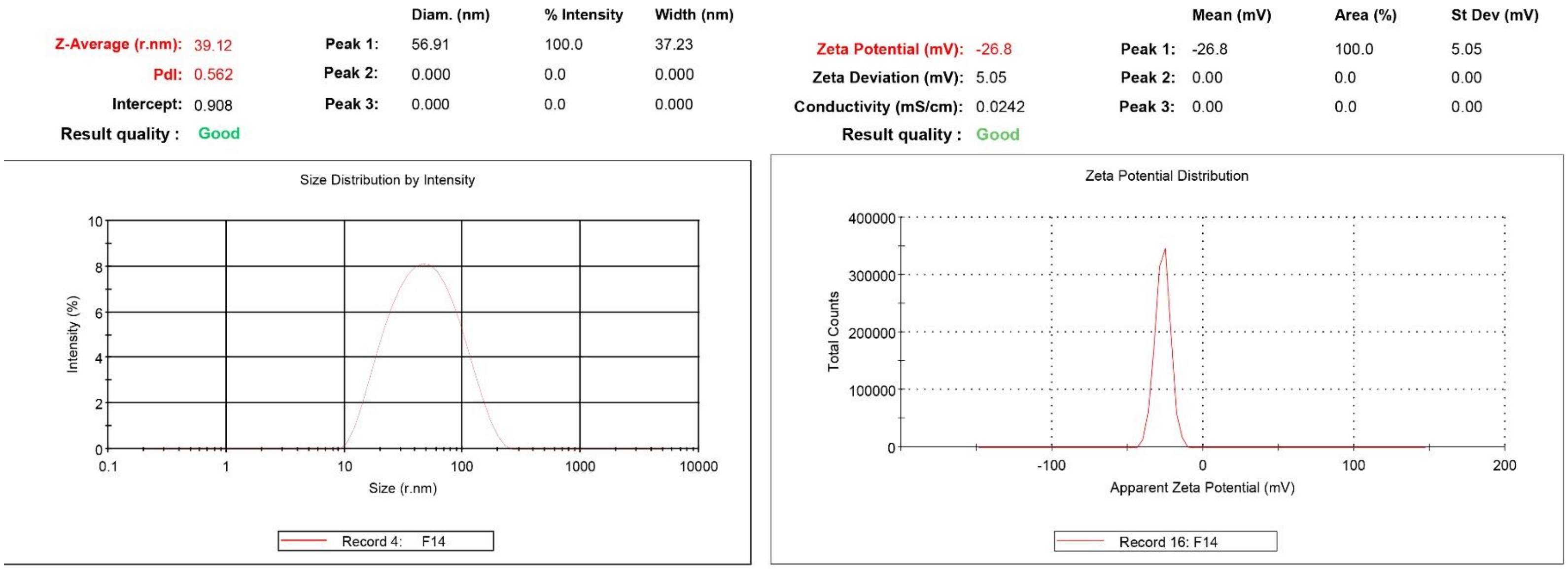


**Figure S3:** Droplet size (*39.12 nm with PdI 0.562*) and zeta potential (-*26.8 mV*) of TMQ-NE [F14]
